# ZTE MRI improves detection of calcific deposits and differentiation between resorptive and formative phases in calcific tendinitis of shoulder

**DOI:** 10.1038/s41598-025-91983-0

**Published:** 2025-04-09

**Authors:** Ji Young Hwang, Hyein Yun, In Na Yoon, Min-Yung Chang, Minseong Kang, Sang-Jin Shin, Moon jung Hwang

**Affiliations:** 1 Department of Radiology, College of Medicine, Ewha Womans University, Ewha Womans University Seoul Hospital, 260, Gonghang-daero, Gangseo-gu, Seoul, 07804 Republic of Korea; 2 Department of Orthopaedic Surgery, College of Medicine, Ewha Womans University, Ewha Womans University Seoul Hospital, 260, Gonghang-daero, Gangseo-gu, Seoul, 07804 Republic of Korea; 3 Advanced Medical Imaging Institute, Korea University Anam Hospital, 73, Goryeodae-ro, Seongbuk-gu, Seoul, 02841 Republic of Korea

**Keywords:** Magnetic resonance imaging, Shoulder, Calcification, Tendons, Tendons, Magnetic resonance imaging, Radiography

## Abstract

The purpose of study was to evaluate the diagnostic value of the ZTE sequence of shoulder MRI in patients with calcific tendinitis by comparing conventional sequences. Seventy-nine patients (43 patients with calcific tendinitis and 36 control group) who underwent both radiography and MRI including ZTE sequence were enrolled in our study. Two radiologists assessed the SNR, image quality, presence, size, multiplicity, shape, margin, signal intensity, composition, and location of calcific deposits on ZTE image, FSPDWI, and T2WI. The diagnostic performance was calculated with radiography as the standard of reference. The inter-reader agreement and differentiation of the resorptive phase and formative phase on ZTE were compared to that on FSPDWI and T2WI. A total 59 calcific deposits in 43 patients were found on radiography and classified into type 1 resorptive phases (*n* = 20) and type 2 formative phases (*n* = 39). ZTE image quality was for diagnostic use with substantial inter-reader agreement. Sensitivity and detection rate of ZTE image was better than those of FSPDWI and T2WI. The inter-reader agreement of ZTE image was equal to FSPDWI and better than T2WI. The features of calcific deposits on ZTE images were significantly different between two phases. In the resorptive phase, a greater percentage of calcific deposits showed irregular shapes, ill-defined margin, heterogeneous compositions on ZTE images. In conclusion, ZTE sequence may provide useful information for the diagnosis of calcific tendinitis of the shoulder.

## Introduction

Calcific tendinitis of the shoulder is characterized by calcific deposits in the rotator cuff tendon or subacromial bursa^1^. Calcific deposits are most frequently located in the supraspinatus tendon (SST), followed by the infraspinatus tendon (IST), and are rarely found in the subscapularis tendon (SSC) and teres minor tendon (TM)^2^.

Early identification and localization of the calcific deposits associated with tendons are important because the calcific deposits in the rotator cuff tendon may cause shoulder pain^3^. Calcific tendinitis has three stages, and the second stage has two phases: (i) the precalcific stage, with fibrocartilaginous tendon transformation, and (ii) the calcific stage, with calcium crystal deposition (formative phase) followed by resorption due to macrophage activation (resorptive phase). During this stage, edema and the extravasation of calcium crystals in the subacromial bursa occur, leading to increased intratendinous pressure and pain. (iii) In the postcalcific stage, tendon matrix remodeling by fibroblasts occurs, and calcium crystals are replaced by granulation tissue, leading to complete tendon healing^4,6^.

DePalma and Kruper classified two types of calcific deposits observed via radiography. According to their classification, type 1 calcific deposits are poorly defined, fluffy, and amorphous which are usually associated with acute pain and can be considered to be in the resorptive phase. Conversely, type 2 calcific deposits are well-defined, and homogeneous which are usually associated with subacute or chronic pain and can be considered to be in the formative phase^7^. In the resorptive phase of calcific tendinitis, several conservative treatments could be indicated and result in a good treatment response^1^.

Radiography is the most frequently used method for identifying calcific deposits. In addition to radiography, ultrasonography (US), magnetic resonance imaging (MRI), and computed tomography (CT) can be used to evaluate calcific deposits. US has been reported to be a highly accurate imaging modality for assessing calcific deposits on the rotator cuff, with a sensitivity of 94%, specificity of 99%, accuracy of 99%, positive predictive value of 95%, and negative predictive value of 99%^8^. Conversely, conventional MRI has been found to have lower diagnostic performance, with a sensitivity of 47.5–59.1%, specificity of 59.1–98.7%, accuracy of 87.1–90.1%, positive predictive value of 80.0–92.1%, and negative predictive value of 36.4–89.8%^9^. There are both advantages and disadvantages for the diagnosis of calcific tendinitis with each imaging modality.

The zero echo time (ZTE) MRI sequence enables the acquisition of signals from tissues with very short T2 relaxation, including bone. In previous studies, investigators reported the diagnostic usefulness of ZTE for identifying bone structures in the skull^10^, evaluating osseous shoulder imaging^11^, assessing the glenoid bone^12^, and evaluating the osseous morphology of the hip^13^. However, to the best of our knowledge, there is only one study on the clinical applicability of ZTE shoulder MRI for the diagnosis of calcific tendinitis of the shoulder. They reported that the addition of ZTE to standard MRI compared to standard MRI increased the diagnostic performance for calcific tendinitis, with a sensitivity of 75.4–77%, specificity of 96.6–98.7%, accuracy of 92.2–94.2%, positive predictive values of 85.2–94%, and negative predictive values of 93.7–94.3%^6^.

The purpose of this study was to evaluate the feasibility of the ZTE MRI sequence for the diagnosis of calcific tendinitis of the shoulder in comparison with conventional MRI sequences. We analyzed not only the inter-reader agreement and imaging features between ZTE and conventional MRI sequences but also the ability of ZTE to differentiate between calcific deposits in the resorptive phase and those in the formative phase.

## Methods

### Patient selection

The institutional review board approved this study and waived the requirement for informed consent owing to the retrospective design of the study (Ewha Womans University Seoul Hospital, No. SEUMC 2022-10-044). The methods of study have been performed in accordance with the Declaration of Helsinki and the STROBE (Strengthening the Reporting of Observational Studies in Epidemiology) guidelines. From February 2019 until May 2022, 825 patients with shoulder pain having radiography and MRI with ZTE sequence were included in our study. Patients were excluded for this study because of previous shoulder surgery (*n* = 212), infections (*n* = 23), tumors (*n* = 11), fractures and dislocation (*n* = 157), incomplete data sets (*n* = 343). The final study population was composed of 79 patients. The radiography was considered the standard of reference for calcific tendinitis. All the patients had undergone radiography, including shoulder anteroposterior view, axillary lateral view, and supraspinatus outlet view, and MRI including ZTE image, fat-suppressed proton density weighted image (FSPDWI), and T2 weighted image (T2WI). Medical records were reviewed for age, sex, side, symptom duration, shoulder US examination, treatment for calcific tendinitis, and follow up.

### MRI acquisition and scan parameters

MRI was performed using a 3T system (Signa Architect; GE Healthcare, Milwaukee, Wisconsin) with a shoulder coil.

The shoulder MRI protocols included oblique coronal, oblique sagittal, and transverse FSPDWI; oblique coronal and oblique sagittal T2WI; and coronal ZTE imaging with transverse and sagittal reconstruction using the following scan parameters: (1) FSPDWI; TE, 35.0 ms; TR, 3540 ms; FOV, 160 × 160 mm; slice thickness, 3 mm; flip angle, 111°; matrix size, 320 × 320; image voxel resolution, 0.5 × 0.5 × 3.0 mm3; receiver bandwidth, 50.00 kHz; total number of scans, 27; and scan time, 3 min 26 s; (2) T2WI; TE, 75.0 ms; TR, 4552 ms; FOV, 160 × 160 mm; slice thickness, 3 mm; flip angle, 111°; matrix size, 320 × 320; image voxel resolution, 0.5 × 0.5 × 3.0 mm3; receiver bandwidth, 62.50 kHz; total number of scans, 27; and scan time; 2 min 12 Sect. (3) ZTE imaging; TE, 0.0 ms; TR, 577 ms; FOV, 160 × 160 mm; slice thickness, 0.6 mm; and flip angle, 1°; matrix size, 260 × 260; image voxel resolution, 0.6 × 0.6 × 1.2 mm^3^; receiver bandwidth,50.00 kHz; total number of scans, 176(slice per slab), and scan time; 3 min 49 s.

**Fig. 1:**
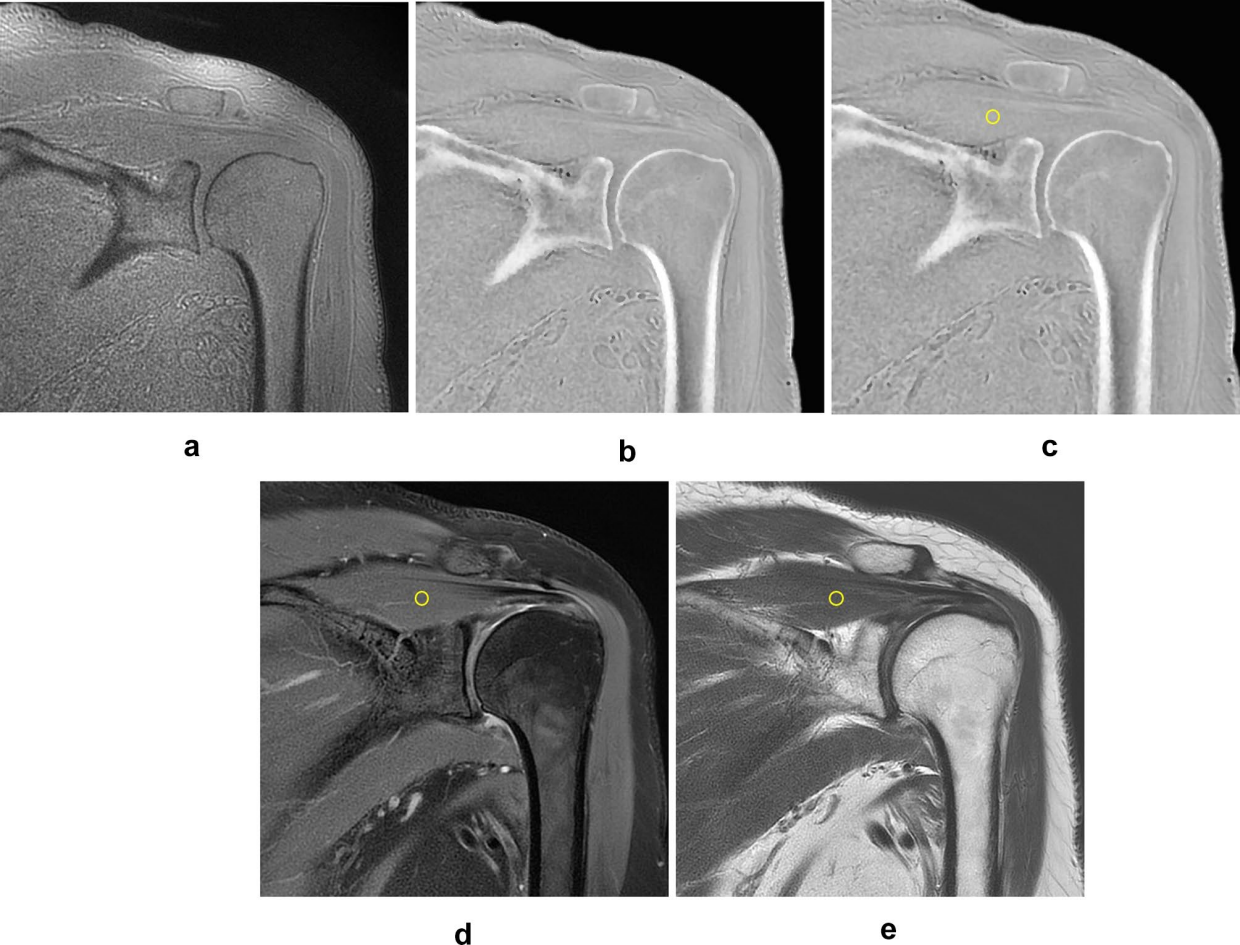
Representative coronal ZTE image of the shoulder joint and ROI placement for SNR measurement. Original ZTE image (**A**) with noisy and black bony cortex and postprocess ZTE image (**B**) after N4 bias-correction and inverse-logarithmic rescaling showing white bony cortex and background suppression in a 73-year-old patient with calcific tendinitis of shoulder. ROIs with a 10 mm^2^ were placed in supraspinatus muscle at spinoglenoid notch level, avoiding central tendon on coronal ZTE image (C), FSPDWI (D), and T2WI (E).

The original ZTE images were noisy due to signal inhomogeneity from coil geometry and variable tissue cross-Sect^11^. CT-like ZTE images were obtained by postprocessing with an N4 bias-correction algorithm and inverse-logarithmic rescaling (Fig. 1).

### Image analysis

Two board-certified musculoskeletal radiologists (with 5 and 20 years of experience) reviewed all the images using the PACS. First, the features of calcific deposits on radiography were analyzed, and a consensus was reached. All calcific deposits were classified as type 1 resorptive phase or type 2 formative phase by DePalma and Kruper on radiography^7^. Two readers were required to attend the shoulder MRI training session which included lectures, discussion for variables, and practice with the example cases to minimize the assessment bias from the different levels of experiences.

After more than 1week washout period, 2 readers independently assessed the signal to noise ratio (SNR), and image quality on ZTE image, FSPDWI, and T2WI. The region of interest (ROI) with a 10 mm^2^ area was meticulously placed in supraspinatus muscle at spinoglenoid notch level, avoiding central tendon (Fig. 1). SNR was calculated using mean signal intensity divided by standard deviation of ROIs. Image quality of MRI was scored as 5-point Likert scale based on clear visualization of shoulder bony structure, visualization of calcification, degree of noise, and absence of artifact: (1) non-diagnostic, (2) unacceptable, (3) acceptable, (4) diagnostic, (5) excellent. We analyzed the presence, size, multiplicity, shape, margin, signal intensity, composition, and location of calcific deposits on each MRI sequences. Other MRI findings including the rotator cuff tear, cartilage thinning, osteophytes, subacromial spur, and inflammation surrounding calcific deposits were also evaluated.

The calcific deposit size was measured manually on each plane, and the largest size of calcific deposits was recorded by comparing all image planes. The shape was classified as irregular, oval, or round. The margin was classified as well-defined or ill-defined. The signal intensity on ZTE images were defined as high when signal intensity of calcific deposits was equal to or greater than that of the bony cortex. The signal intensity on FSPDWI and T2WI was defined as low when the signal intensity of calcific deposits was equal to or lower than that of the tendon. The composition of the calcification was classified as homogeneous or heterogeneous. The locations of calcific deposits such as SST, IST, SSC, and TM were recorded. In case of US examination, size and US features were described. In case of follow up, radiography was evaluated for the presence and size reduction of calcific deposits.

### Statistical analysis

The quantitative variables are presented as means and standard deviations (SDs). Sensitivity, specificity, false positive, false negative, positive predictive value, and negative predictive value for the detection of calcific deposits on ZTE images, FSPDWI, and T2WI were calculated using chi-square tests with radiography as the standard of reference. The inter-reader agreement for image quality and features of calcific deposits on each MRI sequence was analyzed using kappa (κ) statistics. K values were categorized as poor (0), slight (0.0–0.2), fair (0.21–0.40), moderate (0.41–0.60), substantial (0.61–0.80), or perfect (0.81–1.00)^14^. Spearman correlation coefficients (*rho*) were used to compare calcific deposit size between the radiography and each MRI sequence.

Patient characteristics, the features of calcific deposits on ZTE images, and other MRI findings were compared between the resorptive phase and formative phase using chi-square tests. A *p* value of < 0.05 was considered to indicate statistical significance. All the statistical analyses were conducted using SPSS, version 28.0 (IBM, Armonk, New York) and MedCalc, version 23.1.6 (MedCalc Software Ltd, Belgium).

## Results

### Patient characteristics

Among 79 patients, there were 33 men and 46 women with mean age of 51.95 ± 15.10 years. There were 47 right shoulders and 32 left shoulders.

Forty-three patients (mean age, 56.47 ± 13.41 years, M:F = 16:27) were diagnosed to calcific tendinitis (calcific tendinitis group) and 36 patients (mean age, 46.56 ± 15.40 years, M:F = 17:19) did not have any calcification around shoulder (control group) on radiography. Calcific tendinitis occurred in the right shoulder (*n* = 25) and in the left shoulder (*n* = 18) among 43 patients. There were 3 calcific deposits in 2 patients, 2 in 12 patients, and 1 in 29 patients, thus 59 calcific deposits were found. There were type 1 resorptive phases (*n* = 20) and type 2 formative phases (*n* = 39) among 59 calcific deposits. All patients experienced shoulder pain and the duration of symptoms varied from 1 day to 4 years. The characteristics of the patients are listed in Table 1. US examination was performed in 39 of 43 patients with calcific tendinitis. Conservative treatment for calcific tendinitis was performed in 27 patients with barbotage with injection or bursal injection. Calcific deposits became invisible or reduced in size (*n* = 14), unchanged (*n* = 7) on follow up radiography, and unknown in 6 cases because of follow up loss.

**Table 1:** Characteristics of patients with calcific tendinitis and some MRI findings.

Characteristics	All	Resorptive phase^*^	Formative phase^†^	*p* value
**No. of Patients**	*n* = 43	*n* = 15	*n* = 28	
sex (men/ women)	16 / 27	5 / 10	11 / 17	0.700
median age [years] (range)	56 (31–85)	56.1 (35–85)	56.7 (31–79)	0.889
side (right/left)	25 / 18	9 / 6	16 / 12	0.950
symptom	43 (100%)	15 (34.9%)	28 (65.1%)	NA
median symptom duration [days] (range)	313 (1–1440)	302 (7–1080)	320 (1–1440)	0.870
treatment response on follow up (improve / no change)	14 / 7	9 / 1	5 / 6	0.035**
RCT	9 (20.9%)	3 (7.0%)	6 (14.0%)	0.913
RCT location (SST / IST / SSC / TM)	7 / 1 / 1 / 0	1 / 1 / 1 / 0	6 / 0 / 0 / 0	0.169
RCT type (full thickness/partial thickness)	4 / 5	2 / 1	2 / 4	0.640
cartilage thinning of shoulder joint	3 (7.0%)	1 (2.3%)	2 (4.7%)	0.953
osteophytes of shoulder joint	3 (7.0%)	0 (0%)	3 (7.0%)	0.189
subacromial spur	4 (9.3%)	1 (2.3%)	3 (7.0%)	0.663
**No. of Calcific deposits**	*n* = 59	*n* = 20	*n* = 39	
median size of calcific deposits [mm] (range)	10.03 (2.19–53.85)	15.80 (3.15–53.85)	6.94 (2.19–16.72)	< 0.001**
multiplicity of calcific deposits (1 / 2 / 3)	29 / 12 / 2	11 / 3 / 1	18 / 9 / 1	0.808
inflammation surrounding calcific deposits^‡^	16	13	3	< 0.001**

Data are number of patients or nodules with percentage in parentheses, unless otherwise indicated. ^*^calcific deposits with fluffy, amorphous, unclear margin, ^†^calcific deposits with homogeneous, clear margin, ^‡^ ill-defined signal change surrounding calcification on MRI, RCT = rotator cuff tear, SST = supraspinatus tendon, IST = infraspinatus tendon, SSC = subscapularis tendon, TM = teres minor tendon. ** statistically significant *p*-value < 0.05 between resorptive phase and formative phase.

### Image quality and SNR of ZTE image, FSPDWI, and T2WI

The average mean scores of MRI image quality by both readers were 4.35 in ZTE images, 4.86 in PDWI, and 4.91 in T2WI, which were image quality for diagnostic use. Inter-reader agreements were substantial for image quality of ZTE images (κ = 0.676, 95% CI, 0.549–0.803; *p* < 0.001), PDWI (κ = 0.686, 95% CI, 0.458–0.915; *p* < 0.001), and T2WI (κ = 0.688, 95% CI, 0.406–0.971; *p* < 0.001). The SNR of ZTE images, PDWI, and T2WI were 42.24 ± 16.37, 16.76 ± 5.50, and 6.09 ± 2.14, respectively.

### Diagnostic performances of ZTE image, FSPDWI, and T2WI for diagnosis of calcific tendinitis

On ZTE images, both readers diagnosed correctly in 43 of 43 calcific tendinitis group and made a correctly negative diagnosis in 33 of 36 control group among 79 patients with sensitivity of 100%, specificity of 91.7%, false positive of 8.3%, false negative of 0%, positive predictive value of 93.5%, and negative predictive value of 100% (*p* < 0.001). On PDWI, corresponding values were sensitivity of 93.0%, specificity of 94.4%, false positive of 5.6%, false negative of 7.0%, positive predictive value of 95.2%, and negative predictive value of 91.9% by reader 1 (R1); sensitivity of 93.0%, specificity of 97.2%, false positive of 2.8%, false negative of 7.0%, positive predictive value of 97.6%, and negative predictive value of 92.1% by reader 2 (R2) (*p* < 0.001). On T2WI, corresponding values were sensitivity of 65.1%, specificity of 100%, false positive of 0%, false negative of 34.9%, positive predictive value of 100%, and negative predictive value of 70.6% by R1; sensitivity of 74.4%, specificity of 100%, false positive of 0%, false negative of 25.6%, positive predictive value of 100%, andand negative predictive value of 76.6% by R2 (*p* < 0.001) (Table 2).

**Table 2:** Diagnostic performance of MRI ZTE image, FSPDWI, and T2WI for diagnosis of calcific tendinitis by each reader.

	Reader 1			Reader 2		
	ZTE	FSPDWI	T2WI	ZTE	FSPDWI	T2WI
Sensitivity	100%	93.0%	65.1%	100%	93.0%	74.4%
Specificity	91.7%	94.4%	100%	91.7%	97.2%	100%
Accuracy	96.2%	93.7%	81.0%	96.2%	93.7%	91.1%
Positive predictive value	93.5%	95.2%	100%	93.5%	97.6%	100%
Negative predictive value	100%	91.9%	70.6%	100%	92.1%	76.6%

Data are percentage. MRI = magnetic resonance imaging, ZTE = zero echo time, FSPDWI = fat suppressed proton density-weighted image, T2WI = T2-weighted image.

### Detection rate and imaging features of calcific deposits

**Fig. 2:**
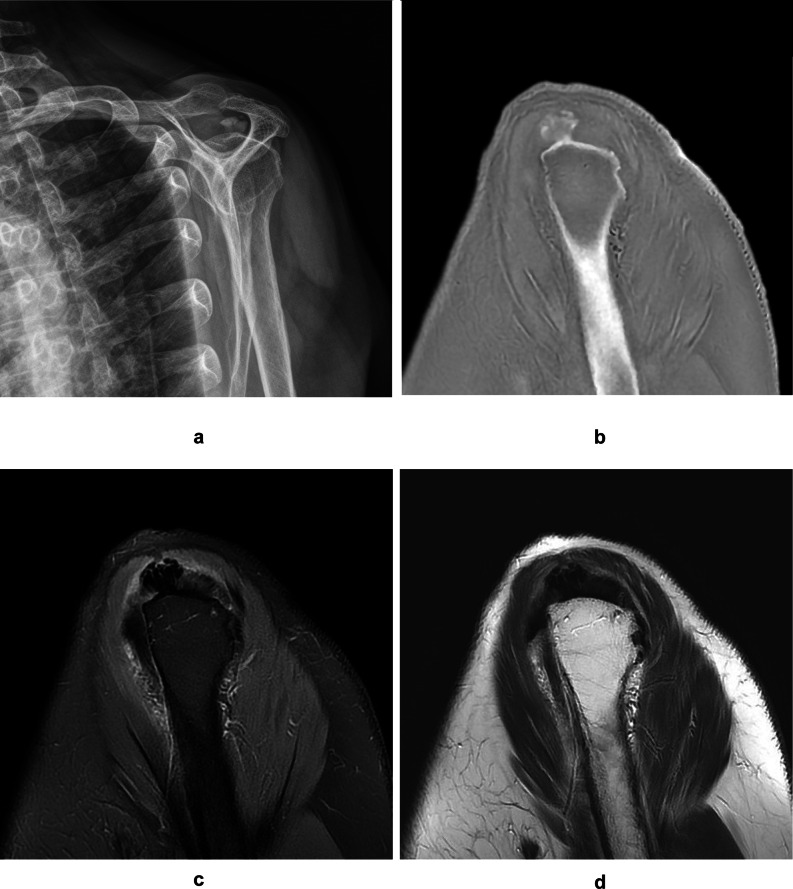
A 85-year-old patient with calcific tendinitis in resorptive phase at supraspinatus tendon of shoulder. Radiography (**A**) showed a poorly defined, fluffy, and amorphous type 1 calcific deposit by Depalma’s classification. Sagittal ZTE image (**B**) showed a irregular shaped, ill-defined calcific deposit with heterogeneous low signal intensity. Sagittal FSPDWI (**C**) showed a irregular shaped calcific deposit with low signal intensity. Note high signal intensity surrounding calcific deposit and subacromial-subdeltoid bursa representing inflammation (arrow). A calcific deposit on sagittal T2WI (**D**) showed similar low signal intensity with surrounding supraspinatus tendon which results in ill-defined margin and smaller size.

The detection rates of calcific deposits were 100% (59 of 59 calcific deposits by R1 and R2) on ZTE images, 84.7% (50 of 59 calcific deposits by R1 and R2) on FSPDWI, and 55.9% (33 of 59 calcific deposits by R1) and 66.1% (39 of 59 calcific deposits by R2) on T2WI. On US, 48 of 59 calcific deposits (81.4%) were detected. The sizes of calcific deposits were 9.21 ± 7.99 on radiography, 8.01 ± 5.06 (by R1) and 8.48 ± 5.52 (by R2) on ZTE images, 7.53 ± 6.77 (by R1) and 8.20 ± 6.84 (by R2) on FSPDWI, 5.42 ± 7.30 (by R1) and 6.36 ± 6.74 (by R2) on T2WI, and 5.08 ± 3.74 on US, respectively. The size of calcific deposits was 11.89 ± 9.62 mm on radiography, 9.96 ± 5.47 mm on ZTE images, 10.28 ± 7.37 mm on FSPDWI, and 9.69 ± 7.35 mm on T2WI among 33 calcific deposits only when observed by R1 in all modalities. The size of calcific deposits was 11.16 ± 8.97 mm on radiography, 9.95 ± 5.67 mm on ZTE images, 10.54 ± 6.84 mm on FSPDWI, and 9.63 ± 6.09 mm on T2WI among 39 calcific deposits only when observed by R2 in all modalities. The size of calcific deposits that were not identified on FSPDWI was 3.50 ± 1.43 mm by R1 and 4.76 ± 4.65 mm by R2. The size of calcific deposits that were not identified on T2WI was 5.82 ± 2.88 by R1 and 5.42 ± 3.35 mm by R2. The features of calcific deposits, including shape, margin, signal intensity, and composition, on each MRI sequence are summarized in Table 3. The most common location of calcium deposits was SST (*n* = 38, 64.4%), followed by IST (*n* = 15, 25.4%) and SSC (*n* = 6, 10.2%). The other MRI findings, including rotator cuff tear, cartilage thinning, osteophytes, subacromial spurs, and inflammation surrounding calcific deposits, are shown in Table 1. There were rotator cuff tears in 9 of 43 patients (20.9%), cartilage thinning in 3 of 43 patients (7.0%), osteophytes in 3 of 43 patients (7%), subacromial spurs in 4 of 43 patients (9.3%), and inflammation surrounding calcific deposits in 16 of 59 calcific deposits (27.1%). Among those MRI findings, rotator cuff tear, cartilage thinning, and inflammation surrounding calcific deposits were not observed on ZTE images but were observed on FSPDWI and T2WI (Fig. 2). The US features of calcific tendinitis were irregular shaped hyperechoic lesion with posterior shadowing (*n* = 14) and hyperechoic clusters without posterior shadowing (*n* = 34).

**Table 3:** Differentiation of features of calcific deposits on MRI between type 1 resorptive phase and type 2 formative phase.

Features of calcific deposits	Reader 1				Reader 2				
	Resorptive phase	Formative phase	Total	*p*-value		Resorptive phase	Formative phase	Total	*p*-value
**Shape** (irregular / oval / round)									
ZTE FSPDWI T2WI	17 / 3 / 0 16 / 4 / 0 13 / 4 / 1	8 / 23 / 8 4 / 21 / 5 2 / 10 / 3	25 / 26 / 8 20 / 25 / 5 15 / 14 / 4	< 0.001** < 0.001** 0.003**		19 / 1 / 0 18 / 2 / 0 17 / 2 / 0	9 / 25 / 5 10 / 20 / 0 9 / 11 / 0	28 / 26 / 5 28 / 22 / 0 26 / 13 / 0	< 0.001** 0.001** 0.003**
**Margin** (well-defined / ill-defined^*^)									
ZTE FSPDWI T2WI	10 / 10 14 / 6 5 / 13	33 / 6 19 / 11 2 / 13	43 / 16 33 / 17 7 / 26	0.005** 0.629 0.319		10 / 10 8 / 12 6 / 13	33 / 6 15 / 15 8 / 12	43 / 16 23 / 27 14 / 25	0.005** 0.491 0.588
**SI** (high ^†^/ low^‡^)									
ZTE FSPDWI T2WI	11 / 9 1 / 19 0 / 18	36 / 3 1 / 29 0 / 15	47 / 12 2 / 48 0 / 33	< 0.001** 0.771 0.602		12 / 8 0 / 20 0 / 19	36 / 3 2 / 28 0 / 20	48 / 11 2 / 48 0 / 39	0.003** 0.243 0.873
**Composition** (homogeneous / heterogeneous)									
ZTE FSPDWI T2WI	7 / 13 15 / 5 12 / 6	34 / 5 28 / 2 14 / 1	41 / 18 43 / 7 26 / 7	< 0.001** 0.070 0.066		8 / 12 12 / 8 12 / 7	33 / 6 26 / 4 19 / 1	41 / 18 38 / 12 31 / 8	< 0.001** 0.032** 0.015**

Data are number of cases. MRI = magnetic resonance imaging, ZTE = zero echo time, FSPDWI = fat suppressed proton density-weighted image, T2WI = T2 weighted image, ^*^ Indistinct, microlobulated, spiculated margin, SI = signal intensity used in MRI, ^†^ Equal or higher than bony cortex on ZTE, ^‡^ Lower than bony cortex on ZTE. ** statistically significant *p*-value < 0.05 between resorptive phase and formative phase.

The inter-reader agreement of the features of calcific deposits was ‘substantial’ to ‘perfect’ on ZTE images (κ = 0.755 ~ 0.946; *p* < 0.001) and on FSPDWI (κ = 0.648 ~ 0.863; *p* < 0.001), while it was ‘moderate’ to ‘substantial’ on T2WI (κ = 0.500 ~ 0.699; *p* < 0.001) (Table 4).

**Table 4:** Comparison of inter-reader agreement for imaging features of calcific tendinitis among MRI sequences between two readers.

Imaging features of calcific deposits	Inter-reader agreement (kappa value)	95% confidence interval	*p* value
**ZTE**			
Shape (irregular, oval, round)	0.755	0.614–0.896	< 0.001
Margin (well-defined, ill-defined)	0.914	0.797–1.000	< 0.001
Signal intensity (high, low)	0.946	0.841–1.000	< 0.001
Composition (homogeneous, heterogeneous)	0.920	0.811–1.000	< 0.001
Location (SST, IST, SSC, TM)	0.843	0.689–0.997	< 0.001
**FSPDWI**			
Shape (irregular, oval, round)	0.666	0.504–0.829	< 0.001
Margin (well-defined, ill-defined)	0.648	0.488–0.808	< 0.001
Signal intensity (high, low)	0.686	0.415–0.956	< 0.001
Composition (homogeneous, heterogeneous)	0.779	0.640–0.918	< 0.001
Location (SST, IST, SSC, TM)	0.863	0.767–0.958	< 0.001
**T2WI**			
Shape (irregular, oval, round)	0.568	0.402–0.735	< 0.001
Margin (well-defined, ill-defined)	0.500	0.321–0.679	< 0.001
Signal intensity (high, low)	0.647	0.453–0.841	< 0.001
Composition (homogeneous, heterogeneous)	0.603	0.415–0.791	< 0.001
Location (SST, IST, SSC, TM)	0.699	0.541–0.857	< 0.001

MRI = magnetic resonance imaging, ZTE = zero echo time, FSPDWI = fat suppressed proton density-weighted image, T2WI = T2-weighted image, SST = supraspinatus tendon, IST = infraspinatus tendon, SSC = subscapularis tendon, TM = teres minor tendon, SD = standard of deviation.

The size of calcific deposits on radiography was positively correlated with that on MRI (*rho* = 0.844 ~ 0.888 on ZTE image, *rho* = 0.771 ~ 0.878 on FSPDWI, and *rho* = 0.664 ~ 0.698 on T2WI; *p* < 0.001). The size of calcific deposits on ZTE images was positively correlated with that on FSPDWI (*r* = 0.890 ~ 0.946; *p* < 0.001) and that on T2WI (*r* = 0.682 ~ 0.757; *p* < 0.001). The size of calcific deposits on FSPDWI was correlated with that on T2WI (*r* = 0.713 ~ 0.836; *p* < 0.001).

### Comparison of calcific tendinitis between the resorptive phase and formative phase

There were no statistically significant differences in the rotator cuff tear, cartilage thinning, osteophytes, or subacromial spur between two phases. However, size and surrounding inflammation of calcific deposits were significantly different between two phases. The median size of calcific deposits was larger in the resorptive phase (15.80 mm) than in formative phase (6.94 mm) (*p* < 0.001). Inflammation surrounding calcific deposits was more frequently observed in the resorptive phase (*n* = 13 of 20, 65%) than in the formative phase (*n* = 3 of 39, 7.6%) (*p* < 0.001). The treatment response on follow up was different between two phases (*p* = 0.035). Among 21 patients with follow up radiography, 9 of 10 (90%) patients in resorptive phase and 5 of 11 (45.5%) patients in formative phase showed improvement (Table 1).

**Fig. 3:**
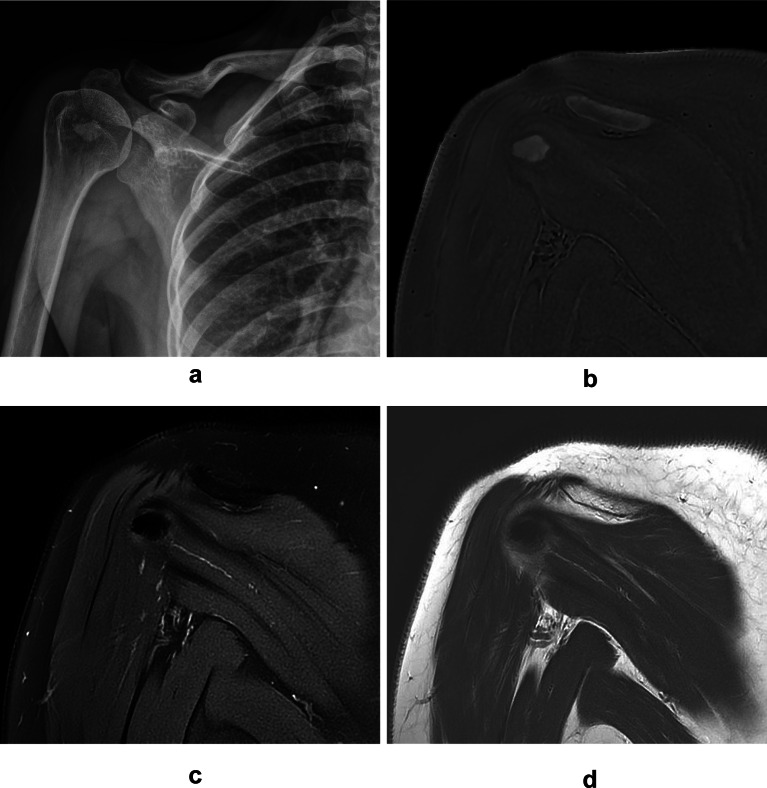
A 40-year-old patient with calcific tendinitis in formative phase at infraspinatus tendon of shoulder. Radiography (**A**) showed a well-defined, homogeneous type 2 calcific deposits by Depalma’s classification. Coronal ZTE image (**B**) showed a oval shaped, well-defined calcific deposit with homogeneous high signal intensity. Coronal FSPDWI (**C**) showed a oval shaped, well-defined calcific deposit with low signal intensity. Coronal T2WI (**D**) showed a smaller sized calcific deposit with indistinct margin and similar low signal intensity with surrounding infraspinatus tendon.

**Fig. 4:**
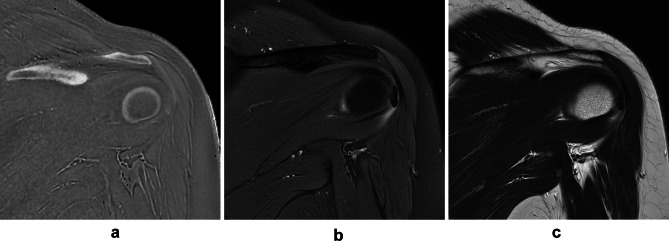
A 46-year-old patient with calcific tendinitis in resorptive phase. Calcific deposit in resorptive phase is very low signal intensity and ill-defined margin on ZTE image (A), so the size of calcific deposit can be underestimated. In contrast, calcific deposit is clearly seen on both FSPDWI (B) and T2WI (C).

Differentiation the resorptive phase from the formative phase using the shape, margin, signal intensity, and composition of calcific deposits were compared on ZTE images, PDWI, T2WI (Table 3). The all 4 features of calcific deposits on ZTE images and the shape on PDWI and T2WI were significantly different between two phases in both readers. The composition of calcific deposits on PDWI and T2WI by R2 was significantly different between two phases as well. The calcific deposits in the resorptive phase had irregular shapes (28.8 to 32.2%) and oval or round shapes (1.7 to 5.1%) (Fig. 2). The calcific deposits in the formative phase had irregular shapes (13.6 to 15.3%) and oval or round shapes (50.9 to 52.6%) (Fig. 3). Approximately 70% of calcific deposits showed well-defined margins, however, the percentage of calcific deposits with ill-defined margins was relatively greater in the resorptive phase (*n* = 10 of 20, 50.0%) than in the formative phase (*n* = 6 of 39, 15.3%). Approximately 80% of calcific deposits exhibited signal intensity equal to or greater than that of the bony cortex on ZTE images, however, the percentage of calcific deposits with some lower signal intensity than in the bony cortex on ZTE images was relatively greater in the resorptive phase (*n* = 8–9 of 20, 40–45%) than in the formative phase (*n* = 3 of 39, 8.3%). The composition of calcific deposits was more heterogeneous (20.3 to 22.0%) than homogeneous (11.9 to 13.6%) in the resorptive phase; while more homogeneous (55.9 to 57.6%) than heterogeneous (8.5 to 10.2%) in formative phase. On the ZTE image, all calcific deposits were visible, but 3 calcific deposits in the resorptive phase were poorly defined by indistinct margins and very low signal intensities, so the sizes of calcific deposits were underestimated (Fig. 4). There were no statistically significant differences in US features between resorptive and formative phases (*p* = 0.712).

## Discussion

Conventional MRI with echo times (TEs) of 1 ms or greater provides little or no detectable signal from cortical bone or calcification, which has an extremely short T2* of approximately 300–500 µs. By using half-sinc radiofrequency (RF) pulses, short hard pulses, or radial or spiral mapping of k-space techniques, nominal TEs of less than 200 µs can be achieved^15^. In the 3D ultrashort echo time (UTE) technique, such a scheme is realized by ramping up a projection gradient as soon as possible after nonselective RF excitation, allowing a TE as low as a few µs. Taking this concept even further, zero echo time can be achieved by switching on the projection gradient before the RF pulse. In this approach, the gradient starts encoding freshly created transverse magnetization without any delay. Therefore, TE is zero, and the full k-space speed is available immediately^16^. Generally, all applications with a focus on tissues and materials, including bone, cartilage, tendon, lung, and teeth with very short T2 or T2*, can potentially benefit from using UTE or ZTE MRI^16^.

In general, conventional MRI may be useful in patients with calcific tendinitis even though there is no detectable signal from calcific deposits because of longer TE^9^. MRI of the shoulder can facilitate not only the diagnosis of concurrent rotator cuff tear and cartilage abnormalities but also inflammation associated with calcific deposits. In addition, MRI has no radiation in contrast to radiography and provides exact information about the location and morphology in three planes. However, higher cost and longer scan time comparing to radiography or ultrasound with lower detection rate, lower sensitivity, higher false negative rate, and negative predictive value were drawback. In our study, calcific deposits showed low signal intensity similar to that of the surrounding rotator cuff tendon on both FSPDWI and T2WI which was similar to previous study^9^. Thus, very small sized calcific deposit was not visualized on either FSPDWI or T2WI. The calcific deposits were invisible more on T2WI than on FSPDWI, because the signal intensity of the surrounding both muscle and tendon was equal to that of calcific deposits on T2WI, while the signal intensity of surrounding muscle was slightly higher than that of calcific deposits on FSPDWI. Because of the limitation of conventional MRIs, the 3D Gradient-echo (GRE) sequence with short T2* and susceptibility-weighted imaging (SWI) with combination of magnitude and phase images were used for diagnosis of calcific deposits and offered better sensitivity and specificity than conventional MRI^9,17^.

Compared with conventional MRI such as FSPDWI and T2WI, the detection rate of calcific deposits was the highest on ZTE image (100%), followed by FSPDWI and T2WI (84.7%, and 55.9–66.1%) with a sensitivity of 100% (vs. 93% and 65.1–74.4%,), a specificity of 91.7% (vs. 94.4–97.2% and 100%), a positive predictive value of 93.5% (vs. 95.2–97.6% and 100%), and a negative predictive value of 100% (vs. 91.9–92.1% and 70.6–76.6%.) The mean size of the calcific deposits was measured in the following order: radiography, ZTE image, FSPDWI, and T2WI. The magnification caused by the long distance between the radiography tube and the patient, as well as the thick slab on the projection image of radiography, may explain why the mean calcium deposit size is largest on radiography is largest on radiography^9^. ZTE demonstrated ‘substantial’ to ‘perfect’ inter-reader agreement similar to that of PDWI and superior to that of T2WI in identifying calcific deposits and assessing features of calcific deposits in our study. ZTE image quality was comparable to PDWI and T2WI with a score more than 4 (diagnostic for use). Inter-reader agreement of image quality was ‘substantial’ on all sequences.

However, there were several challenges in clinical application of ZTE in calcific tendinitis. First, the diagnosis of concurrent rotator cuff tear, cartilage abnormalities, and inflammation associated with calcific deposits by conventional MRI was not made by ZTE. Second, although most of calcific deposits were visible, the size of some calcific deposits in the resorptive phase could be underestimated. Third, potential pitfalls of ZTE have been reported. Collagen-rich structures such as the tendon, ligament, and labrum and mucinous, hemorrhagic joint or bursa or tendon sheath fluid may be hyperintense on ZTE, which could be misinterpreted as calcific tendinitis or bursitis^6^. Fourth, ZTE sequence is not currently available in all MRI scanner, so software upgrade or specific MRI equipment for ZTE sequence might be required. Fifth, higher cost comparing to radiography or ultrasound and additional scan time and postprocessing time should be considered. These limitations might be barriers to a widespread application of ZTE sequences for calcific tendinitis.

The differentiation between the resorptive phase and formative phase is important because the treatment strategy and response differ according to the phase of calcific deposits. Calcific tendinitis is generally considered as a self-resolving disease^18^. Conservative treatment is the primary approach for managing calcific tendinitis in the resorptive phase. Nonsteroidal anti-inflammatory drugs are used to manage acute pain associated with calcific tendinitis. The US-guided barbotage technique can help reduce pain through the decompression effects of aspirating and flushing calcified deposits. Extracorporeal shock wave therapy (ESWT) is another effective treatment for pain relief. In patients who do not respond to conservative treatment after 6 months, surgical intervention should be considered. Typically, acute pain associated with calcific tendinitis in the resorptive phase responds well to conservative treatment. However, chronic pain often requires surgical intervention^19^.

ZTE image can differentiate between the resorptive phase and formative phase of calcific deposits more accurately than FSPDWI and T2WI. In our study, calcific deposits in the resorptive phase had a greater percentage of irregular shapes, relatively ill-defined margins, relatively low signal intensity, and heterogeneous composition, while calcific deposits in the formative phase had a greater percentage of oval or round shapes, relatively well-defined margins, relatively high signal intensity, and homogeneous composition on the ZTE image. The shape, margin, signal intensity, and composition of calcific deposits were significantly different between two phases on ZTE images (*p* < 0.0001). In contrast, only the shape and composition were different between two phases on PDWI and T2WI (*p*= 0.001 ~ 0.032). In a previous study, the calcific deposits in type C resorptive phase was missed with ZTE image in 19.3 to 23.1% of cases^6^, which comprised false negative results. In our study, most of calcific deposits in formative phase were visible clearly, but a few calcific deposits in the resorptive phase showed indistinct margins and very low signal intensity on the ZTE image. Therefore, the size of calcific deposits in resorptive phase was underestimated on the ZTE image. The inflammation surrounding calcific deposits on FSPDWI was frequently seen in the resorptive phase than in the formative phase (*p* < 0.0001). The treatment response on follow up was different between two phases (*p* = 0.035) and more patients in resorptive phase showed improvement than patients with formative phase.

US can also be used to classify calcific deposits into resorptive and formative phases. In the resorptive phase, hyperechoic areas are relatively reduced, and the posterior acoustic shadow is either reduced or not visible. Conversely, in the formative phase, hyperechoic areas and a clear posterior acoustic shadow can be observed^8^.

Our study has several limitations. First, our sample size was relatively small which may affect the statistical power of study results. Second, we did not confirm calcific deposits by surgery, so radiography was used as the reference of standard. Third, other imaging modalities such as GRE, SWI, and CT were not analyzed as comparative diagnostic tools for patients with calcific tendinitis, although US was analyzed in some patients. Fourth, short-term and long- term evolution of calcific tendinitis on MRI including ZTE image were not evaluated.

In conclusion, ZTE image provided better detection, better diagnostic performance, greater inter-reader agreement, more accurate size prediction, and better differentiation of resorptive phase and formative phase of calcific deposits as compared with FSPDWI and T2WI. ZTE image may offer valuable diagnostic information in patients with calcific tendinitis of the shoulder as conjunction with FSPDWI and T2WI. Future studies with larger sample size might be required to evaluate how ZTE image could affect treatment decision-making and patient outcome.

## Data Availability

Data are provided within the manuscript. For researchers who may wish to have access to other imaging data of this study, please contact via the following e-mail and send data inquiry: mshjy@ewha.ac.kr (corresponding author).

## References

[1] Merolla, G., Singh, S., Paladini, P. & Porcellini, G. Calcific tendinitis of the rotator cuff: state of the Art in diagnosis and treatment. *J. Orthop. Traumatol.* **17**, 7–14. 10.1007/s10195-015-0367-6 (2016). 26163832 10.1007/s10195-015-0367-6 PMC4805635

[2] Louwerens, J. K., Sierevelt, I. N., van Hove, R. P., van den Bekerom, M. P. & van Noort, A. Prevalence of calcific deposits within the rotator cuff tendons in adults with and without subacromial pain syndrome: clinical and radiologic analysis of 1219 patients. *J. Shoulder Elb. Surg.* **24**, 1588–1593. 10.1016/j.jse.2015.02.024 (2015). 10.1016/j.jse.2015.02.024 25870115

[3] Ark, J. W., Flock, T. J., Flatow, E. L. & Bigliani, L. U. Arthroscopic treatment of calcific tendinitis of the shoulder. *Arthroscopy* **8**, 183–188. 10.1016/0749-8063(92)90034-9 (1992). 1637430 10.1016/0749-8063(92)90034-9

[4] Uhthof, H. K. & Sarkar, K. Calcifying tendinitis. *Baillieres Clin. Rheumatol.* **3**, 567–581 (1989). 2624948 10.1016/s0950-3579(89)80009-3

[5] Albano, D. et al. Imaging of calcific tendinopathy around the shoulder: usual and unusual presentations and common pitfalls. *Radiol. Med.* **126**, 608–619. 10.1007/s11547-020-01300-0 (2021). 33151457 10.1007/s11547-020-01300-0 PMC8007494

[6] Puel, U. et al. Zero echo time MRI in shoulder MRI protocols for the diagnosis of rotator cuff calcific tendinopathy improves identification of calcific deposits compared to conventional MR sequences but remains sub-optimal compared to radiographs. *Eur. Radiol.* **33**, 6381–6391. 10.1007/s00330-023-09602-3 (2023). 37014406 10.1007/s00330-023-09602-3

[7] DePalma, A. F. & Kruper, J. S. Long-term study of shoulder joints afflicted with and treated for calcific tendinitis. *Clin. Orthop.* **20**, 61–72 (1961). 13721957

[﻿8] Farin, P. U. & Jaroma, H. Sonographic findings of rotator cuff calcifications. *J. Ultrasound Med.* **14**, 7–14. 10.7863/jum.1995.14.1.7 (1995). 7707483 10.7863/jum.1995.14.1.7

[9] Zubler, C. et al. MR arthrography in calcific tendinitis of the shoulder: diagnostic performance and pitfalls. *Eur. Radiol.* **17**, 1603–1610. 10.1007/s00330-006-0428-6 (2007). 17036154 10.1007/s00330-006-0428-6

[10] Cho, S. B. et al. Clinical feasibility of zero TE skull MRI in patients with head trauma in comparison with CT: A Single-Center study. *Am. J. Neuroradiol.* **40**, 109–115 (2019). 30545839 10.3174/ajnr.A5916 PMC7048604

[11] Breighner, R. E. et al. Technical developments: zero echo time imaging of the shoulder: enhanced osseous detail by using MR imaging. *Radiology* **286**, 960–966. 10.1148/radiol.2017170906 (2018). 29117482 10.1148/radiol.2017170906

[12] de Mello, R. A. F. et al. Three-Dimensional zero echo time magnetic resonance imaging versus 3-Dimensional computed tomography for glenoid bone assessment. *Arthroscopy: J. Arthroscopic Relat. Surg.* **36**, 2391–2400. 10.1016/j.arthro.2020.05.042 (2020). 10.1016/j.arthro.2020.05.042 PMC7483823 32502712

[13] Breighner, R. E., Bogner, E. A., Lee, S. C., Koff, M. F. & Potter, H. G. Evaluation of osseous morphology of the hip using zero echo time magnetic resonance imaging. *Am. J. Sports Med.* **47**, 3460–3468. 10.1177/0363546519878170 (2019). 31633993 10.1177/0363546519878170

[14] Kundel, H. L. & Polansky, M. Measurement of observer agreement. *Radiology* **228**, 303–308. 10.1148/radiol.2282011860 (2003). 12819342 10.1148/radiol.2282011860

[15] Du, J. et al. Qualitative and quantitative ultrashort echo time (UTE) imaging of cortical bone. *J. Magn. Reson.* **207**, 304–311. 10.1016/j.jmr.2010.09.013 (2010). 20980179 10.1016/j.jmr.2010.09.013

[16] Weiger, M. & Pruessmann, K. P. in *Encyclopedia of Magnetic Resonance* (2012).

[17] Nörenberg, D. et al. Diagnosis of calcific tendonitis of the rotator cuff by using Susceptibility-weighted MR imaging. *Radiology* **278**, 475–484. 10.1148/radiol.2015150034 (2016). 26347995 10.1148/radiol.2015150034

[18] Compagnoni, R. et al. Long-term evolution of calcific tendinitis of the rotator cuff: clinical and radiological evaluation 10 years after diagnosis. *J. Orthop. Traumatol.* **22**, 42. 10.1186/s10195-021-00604-9 (2021). 34698958 10.1186/s10195-021-00604-9 PMC8548447

[19] Kim, M. S., Kim, I. W., Lee, S. & Shin, S. J. Diagnosis and treatment of calcific tendinitis of the shoulder. *Clin. Shoulder Elb.* **23**, 210–216. 10.5397/cise.2020.00318 (2020). 33330261 10.5397/cise.2020.00318 PMC7726362

